# Ceramic Toughening Strategies for Biomedical Applications

**DOI:** 10.3389/fbioe.2022.840372

**Published:** 2022-03-07

**Authors:** Rushui Bai, Qiannan Sun, Ying He, Liying Peng, Yunfan Zhang, Lingyun Zhang, Wenhsuan Lu, Jingjing Deng, Zimeng Zhuang, Tingting Yu, Yan Wei

**Affiliations:** ^1^ Department of Orthodontics, Peking University School and Hospital of Stomatology, Beijing, China; ^2^ National Engineering Laboratory for Digital and Material Technology of Stomatology and Beijing Key Laboratory of Digital Stomatology, Beijing, China; ^3^ Department of Geriatric Dentistry, Peking University School and Hospital of Stomatology, Beijing, China

**Keywords:** ceramic, biomedical, fracture, toughening, reinforcing phase, surface modification, manufacture, toughening mechanism

## Abstract

Aiming at shortage of metal materials, ceramic is increasingly applied in biomedicine due to its high strength, pleasing esthetics and good biocompatibility, especially for dental restorations and implants, artificial joints, as well as synthetic bone substitutes. However, the inherent brittleness of ceramic could lead to serious complications, such as fracture and disfunction of biomedical devices, which impede their clinical applications. Herein, several toughening strategies have been summarized in this review, including reinforcing phase addition, surface modification, and manufacturing processes improvement. Doping metal and/or non-metal reinforcing fillers modifies toughness of bulk ceramic, while surface modifications, mainly coating, chemical and thermal methods, regulate toughness on the surface layer. During fabrication, optimization should be practiced in powder preparation, green forming and densification processes. Various toughening strategies utilize mechanisms involving fine-grained, stress-induced phase transformation, and microcrack toughening, as well as crack deflection, bifurcation, bridging and pull-out. This review hopes to shed light on systematic combination of different toughening strategies and mechanisms to drive progress in biomedical devices.

## 1 Introduction

With the aging of the population, the demand for maintaining the quality of life is in needed worldwide. The tooth loss (Zitzmann et al., 2007), symptomatic osteoarthritis (Pivec et al., 2012; Hunter and Bierma-Zeinstra, 2019), as well as bone defects and dysfunction (Carrington, 2005; Olshansky et al., 2005) have become global health care problems, which lead to growing markets for high-quality biomedical devices (Zitzmann et al., 2007; Kim et al., 2008; Chevalier and Gremillard, 2009; Carr et al., 2012; Patel et al., 2015). Large amount of these devices are made of metal but their metal-color (Bayne et al., 2019), high wear rates of bearing components and large amount of ion release (Chevalier and Gremillard, 2009) greatly impede their clinical applications. Thus, benefited from its excellent mechanical, esthetic (Bayne et al., 2019) and biocompatible properties (Gao et al., 2014; Ruiz Henao et al., 2021), ceramic becomes potential candidate for biomedical applications, such as dental restorations (Li et al., 2014; Spitznagel et al., 2018), dental implants (Kohal et al., 2008; Saadaldin and Rizkalla, 2014), heads and cups of joint replacements (Rahaman et al., 2007), as well as bone fillers and scaffolds (Ma et al., 2018) for tissue engineering. Although ceramic materials show their huge potential and preponderance, high brittleness of ceramic restrains its biomedical applications, which might result in severe clinical complications, such as fracture (Benaqqa et al., 2005; Koo et al., 2014; Morimoto et al., 2016; Kong et al., 2019). Failures of ceramic mostly happen in the absence of extrinsic shielding mechanisms, where fracture invariably occurs catastrophically by cohesive bond breaking at the crack tip with a resulting very low intrinsic toughness of roughly 1–3 MPa m^1/2^ (Launey and Ritchie, 2009). Therefore, the extreme brittleness of ceramic materials highlights the importance of ceramic toughening.

Toughness of materials could be thought as the ability to dissipate deformation energy without crack propagation (Launey and Ritchie, 2009). One of the widely recognized methods of computing toughness, denoted here as τ, is by the area under the engineering stress σ vs. engineering strain ε curve determined in a tensile test (Sehaqui et al., 2013). Thus

$$\tau =\overset{\varepsilon_{b}}{\underset{0}{\int }}\sigma d\varepsilon$$ (1)

Where ε_b_ is the elongation at break. The term “toughness” is also used to the critical outcome of impact testing, namely the energy required to generate fracture at high rates of force application (Bai et al., 2012). Thus, there is fracture toughness, notch toughness, determined in impact testing for a predetermined geometry of the notch and testing methods, or else drop impact toughness (Brostow et al., 2015). Fracture toughness is the focus of this review, and a variety of testing methods have been proposed, such as indentation fracture (IF) (Evans and Charles, 1976; Lawn et al., 1980; Anstis et al., 1981), single-edge notched beam (SENB) (Damani et al., 1996), single-edge precracked beam (SEPB) (Cesar et al., 2005), single-edge V-notched beam (SEVNB), chevron notch (CN), surface crack in flexure (SCF) and double cantilever beam (DCB) methods (Wang et al., 2017).

Crack growth is promoted ahead of the crack tip by intrinsic microstructural damage, and impeded by extrinsic mechanisms acting primarily behind the crack tip, which serve to “shield” the crack tip from the far-field driving forces (Ritchie, 1988). Toughness can be enhanced by increasing the microstructural resistance, such as by changing the nature and distribution to suppress damage in the form of microcracking or microvoid formation ahead of the crack tip, which is termed intrinsic toughening. However, this approach is largely ineffective with brittle materials such as ceramic (Evans, 1990), which invariably must rely on extrinsic toughening. Extrinsic toughening involves microstructural mechanisms that act primarily behind the crack tip to effectively reduce the crack-driving force actually experienced at the crack tip; this is termed crack-tip shielding and can occur by such mechanisms as *in situ* phase transformations and crack bridging (Launey and Ritchie, 2009). Brittle materials, such as ceramics, are invariably toughened with extrinsic mechanisms (Evans, 1990; Becher, 1991), which depend on crack size and to some degree specimen geometry. A principle manifestation of this crack-size dependent fracture behavior is resistance-curve (R-curve) toughness behavior where the crack driving force to sustain cracking increases with crack extension. Extrinsic toughening mechanisms mainly affect the crack growth, while have little effect on crack initiation.

**FIGURE 1:**
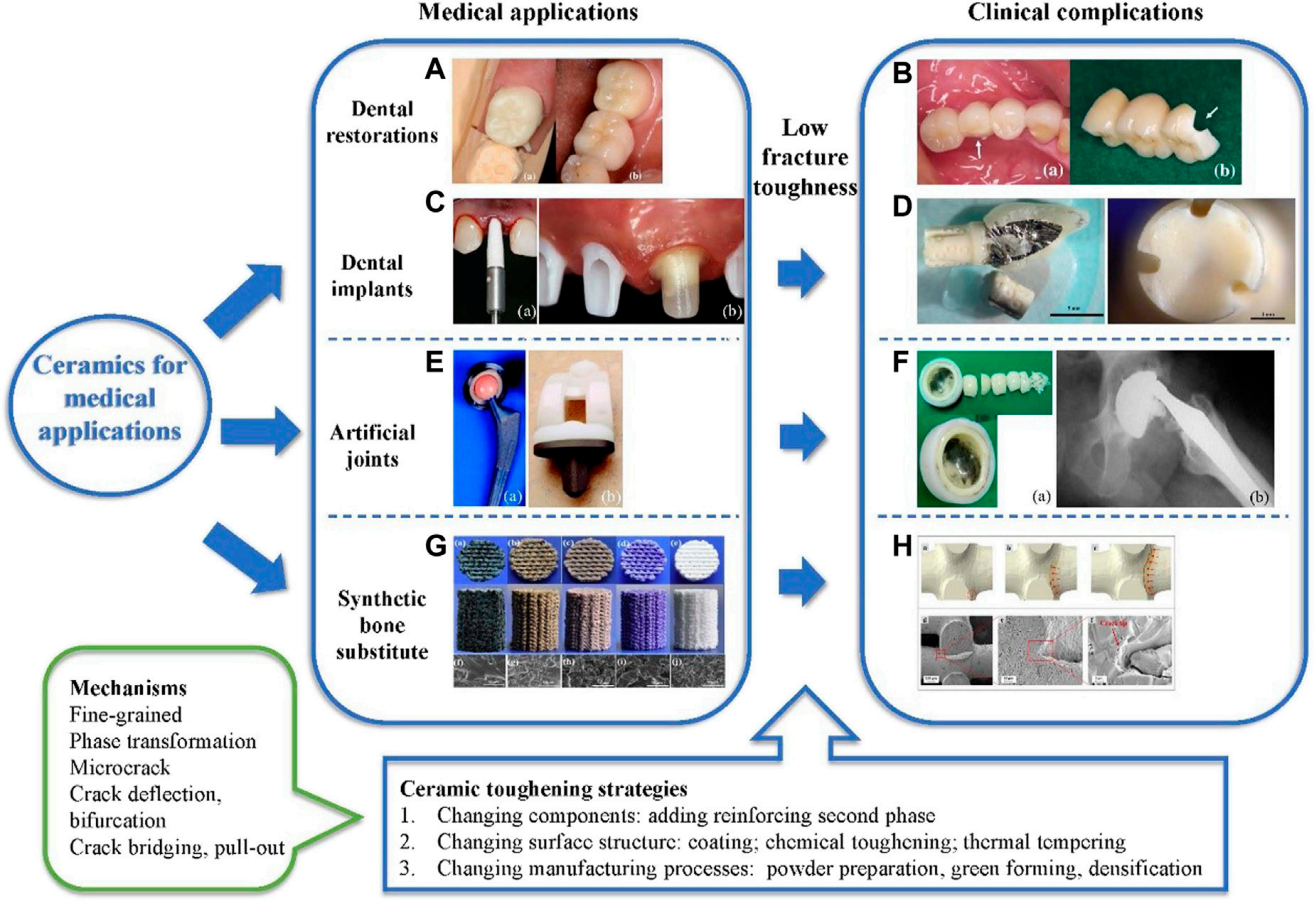
Medical applications of ceramics, corresponding clinical complications due to the low fracture toughness of ceramics, as well as toughening strategies and underlying mechanisms. **(A)** Fully contoured zirconia crown, **(a)** before polishing and **(b)** from the occlusal view (second lower left molar) (Miyazaki et al., 2013). **(B)**, **(a)** Chipping of the ceramic veneer; **(b)** Framework fracture in the second upper left molar distal buccal (Miyazaki et al., 2013). **(C)**, **(a)** Insertion of the ceramic implant; **(b)** Ceramic abutments (zirconia) attached to the implants intraorally (Kohal et al., 2008). **(D)** Extracted fractured implant and crown, as well as fractured surface (Kong et al., 2019). **(E)**, **(a)** Prosthetic hip implants with ceramic-on-ceramic (Al_2_O_3_-on-Al_2_O_3_) bearing couple; **(b)** Prosthetic knee implants with ceramic-on-UHMWPE bearing couple (Rahaman et al., 2007). **(F)**, **(a)** Five large pieces of a fractured ceramic head and many small fragments; **(b)** Radiograph showing the fracture of the ceramic femoral head (Toran et al., 2006; Hwang et al., 2007). **(G)** Photograph of 3D-printed bioactive glass-ceramic scaffolds (Liu et al., 2018). **(H)** Crack initiation and propagation path shown by **(a–c)** XFEM analysis and **(d–f)** FE-SEM images (Entezari et al., 2016). UHMWPE, ultra-high-molecular-weight polyethylene; XFEM, extended finite element method; FE-SEM, field emission scanning electron microscopy.

Biomedical used-ceramic is applied as structural material, where catastrophic fracture is strictly prohibited and might lead to medical malpractice. Therefore, systematic investigation on ceramic toughness and recent progress of its modulation strategies are vastly required. However, there is limited number of reviews concerning ceramic toughening strategies, especially for biomedical applications (Naslain, 2004; Asl et al., 2018; Siddiqui et al., 2018). Therefore, this review provides an overview of crack-tip shielding methods to realize extrinsic toughening for biomedical ceramic (Figure 1), from adding reinforcing second phase (summarized in Table 1), surface modification to manufacturing optimization. And toughening mechanisms are also introduced briefly to deepen understanding of these strategies.

**TABLE 1:** Summary of adding reinforcing second phase in bioceramics.

Classification	Second phase fillers	Volume fraction of fillers	Ceramic matrix	Fabrication techniques	K_lc_ testing method	Maximum K_lc_ (MPa·m^1/2^)	K_Ic_ of control	Mentioned toughening mechanisms	Ref.
Metal Oxide and Metal	Al_2_O_3_ whiskers	2.5 wt%	Al_2_O_3_/3Y-TZP	Conventional sintering (1,500°C, 2h, in air)	IF	6.9 ± 0.8	4.2 ± 0.4 (Pure Al_2_O_3(n)_)	Microcracking, crack deflection, phase transformation toughening	Aguilar-Elguézabal and Bocanegra-Bernal, (2013)
							6.4 ± 0.4 (Pure 3Y-TZP)		
	Al_2_O_3_ platelets	25 wt%	(Ce,Y)-TZP	Sintered at 1,500°C, 2 h	IF	11.3 ± 0.4	7.2 ± 0.5 (3Y-TZP)	Phase transformation toughening, microcracking, denomination coupled toughening, crack deflection, bridging and pull-out	Santos et al. (2021)
	Al_2_O_3_ + SA6	Al_2_O_3_: 0, 5,10, 15 vol%;	1Y6Ce-TZP	Pressureless sintering	IF	12.5	Not mentioned	Phase transformation toughening, crack deflection, crack bridging, microcracking	Gommeringer et al. (2019)
		SA6: 15, 10, 5, 0 vol%							
	3Y-TZP	0, 5, 10, 15, 20 wt%	Mica glass ceramic	Two-stage heat treatment sequence	IF	3.6 ± 0.2	0.8 ± 0.2 (mica glass ceramic)	Phase transformation toughening	Gali et al. (2018)
	Nanocrystalline ZrO_2_ (3Y)	CaSiO_3_/ZrO_2_ mole ratio of 80/20 (C8Z2), 60/40 (C6Z4), 40/60 (C4Z6)	Micrometer sized β-CaSiO_3_	SPS	IF	4.08 ± 0.13 (C6Z4)	1.54 ± 0.04 (β-CaSiO_3_)	Few β-CaSiO_3_ transformed into α-CaSiO_3_; the ZrO_2_ phase showed a network structure in the matrix	Long et al. (2008)
	Needle-like ZnOw	1, 3, 5, 10 wt%	Porous CaSO_4_/bioglass scaffolds	SLS	IF	1.67 ± 0.04	Not mentioned	Whisker pull-out, crack bridging, crack deflection, crack branching	Shuai et al. (2016)
	α-Al_2_O_3_	5, 10, 15, 25, 50 wt%	α- CaSiO_3_	Sintered at 1,150°C and 1,250°C, 5 h	IF	0.9 ± 0.1	0.6 ± 0.12 (Pure α- CaSiO_3_)	Forming new phase CaAl_2_O_4_ from reaction of CaSiO_3_ and Al_2_O_3_	Shirazi et al. (2014)
	Cs_2_O-stabilized leucite core particles	0.0–2.0 mol%	Commercial porcelain (VP); synthesized leucite-based porcelain (NP)	Vacuum fired (1,100°C, 20min)	IF	1.42 ± 0.21 (VP)	0.85 ± 0.11 (VP)	Phase transformation toughening	Rasmussen et al. (2004)
						2.15 ± 0.33 (NP)	1.51 ± 0.15 (NP)		
	AgNPs, PtNPs	500 ppm	NS	Fired (vacuum: 730 mmHg, 930°C)	IF	1.42 ± 0.02 (Pt-NS)	1.36 ± 0.03	Greater elasticity of the metal than the matrix glass; generation of hydrostatic stress	Fujieda et al. (2012)
						1.54 ± 0.05 (Ag-NS)			
	AgNPs	100, 200, 500, 1,000 ppm (Ag100, Ag200, Ag500, Ag1000)	NS	Fired (vacuum: 730 mmHg, 930°C)	IF	1.54 ± 0.05 (Ag500)	1.36 ± 0.03	Crack deflection, crack bridging	Uno et al. (2013)
						1.51 ± 0.08 (Ag1000)			
Non-metal	CNT	4 wt%	HA	SPS	IF	2.40 ± 0.60	1.25 ± 0.91 (HA)	Interfacial shear strength and pull-out energy of CNT from the HA matrix	Lahiri et al. (2010)
	GPL	0.81 vol%	ZTA	SPS	SENB	9.05 ± 0.55 (GPL/ZTA, 1,550°C)	6.46 ± 0.65 (Pure ZTA, 1,550°C)	Pull out, crack bridging, crack deflection	Liu et al. (2012)
	GNP	0.5, 1.0, 1.5, 2.0 wt%	CaSiO_3_	HIP	Nanoindentatio*n* experiments	1.77 ± 0.05 (1 wt% GNP)	0.76 ± 0.18 (CaSiO_3_)	Crack bridging, pull-out, branching and deflection	Mehrali et al. (2014)
	GO	0-0.2 wt%	3Y-TZP	Hot-press sintering	IF	8.95 ± 0.59 (0.1 wt% GO)	40.9% lower	Crack deflection, crack bridging, GO put-out	Zhang et al. (2020)
Polymer	PVA fibers	2.5–5 wt%	CPCs	Set at room temperature (24 h), immersed in PBS and placed on a shaker table set to 120 rpm in an incubator (37°C, 72 h)	Three-point flexural test	WOF: 8.7 ± 2.5 KJ m^−2^ (5 wt% PVA fibers)	WOF: 0.020 ± 0.008 KJ m^−2^ (fiber free CPCs)	Fiber bridging, crack deflection, frictional sliding	Kucko et al. (2019)
	PNIPAM-functionalized PVA fibers	2.5 wt%	CPCs	Set at room temperature (12 h), then immersed in PBS (37°C, 3 days)	Three-point flexural tests	WOF> 1500 J m^−2^ (PVA)	WOF< 100J·m^−2^ (CPCs)	Thermoresponsive effect of PNIPAM to increase the fiber-matrix affinity of PVA fibers	Petre et al. (2019)
PICN	Vita Enamic (commercial product)	Not mentioned	SEVNB	1.09 ± 0.05	Not mentioned	Crack deflection, crack bridging	Della Bona et al. (2014)
	49.5 wt% TEGDMA +1 wt% BPO + Bis-GMA	12.3–18.4 wt%	ZrO_2_	Immersing ZrO_2_ networks in the liquid polymer, polymerization under the atmospheric pressure by heat treatment (70°C, 10 h)	SENB	3.69 ± 0.15	Not mentioned	Polymer occupies pore sites, increased densification leads to reduction in the detrimental stress concentration	Li et al. (2017)
Others	Si_3_N_4_	1, 3, 5 wt%	β-CaSiO_3_	Pressureless sintering	SENB	2.3 (3 wt% Si_3_N_4_)	1.1 (pure β-CaSiO_3_)	Not mentioned	Pan et al. (2015)
	L- (+)-Tar	3, 3.5, 4, 4.3 g/ml	Brushite	Set at room temperature (30min), then incubated in distilled water (37°C, 24 h)	Three-point bending method	0.6 ± 0.07 (0.5M L-(+)-Tar at 4.3 powder-to-liquid ratio)	Not mentioned	L- (+)-Tar can decrease the subunit size of brushite crystals	Moussa et al. (2020)
Multi-component	Leucite	0, 6, 15, 22 vol%	Six porcelains^a^	Not mentioned	SEPB	1.23 ± 0.12 (porcelain A)	0.71 ± 0.05 (porcelain Cb); 0.75 ± 0.08 (porcelain V)	Crack deflection around leucite particles and clusters	Cesar et al. (2005)
	Needle-like fluorapatites	0, 1, 3 wt%	Mica-based glass-ceramics	Casting and subsequent heat treatment	IF	3.1 ± 0.3 (Glass 3)	0.8 ± 0.1 (Glass 1)	Frictional bridging and pullout toughening	Xiang et al. (2007)
	MgO	0.5 wt%	Al_2_O_3_-glass composite	Sintering at 1,400°C for 2h, infiltrating the molten glass into the partially sintered alumina compact	SENB	5.12 ± 0.35 (MgO-Al_2_O_3_/glass)	0.58 ± 0.13 (partially sintered Al_2_O_3_)	Crack deflection, crack bowing	Luo et al. (2002)

a Six porcelains: A (Ceramco I/Dentsply), B (Ceramco II/Dentsitply), C (Finesse/Dentsply), D (d.Sign/Ivoclar), Cb (Cerabien/Noritake) and V (Vitadur Alpha/Vita).

## 2 Adding Reinforcing Filler as Second Phase to Ceramic Matrix

**FIGURE 2:**
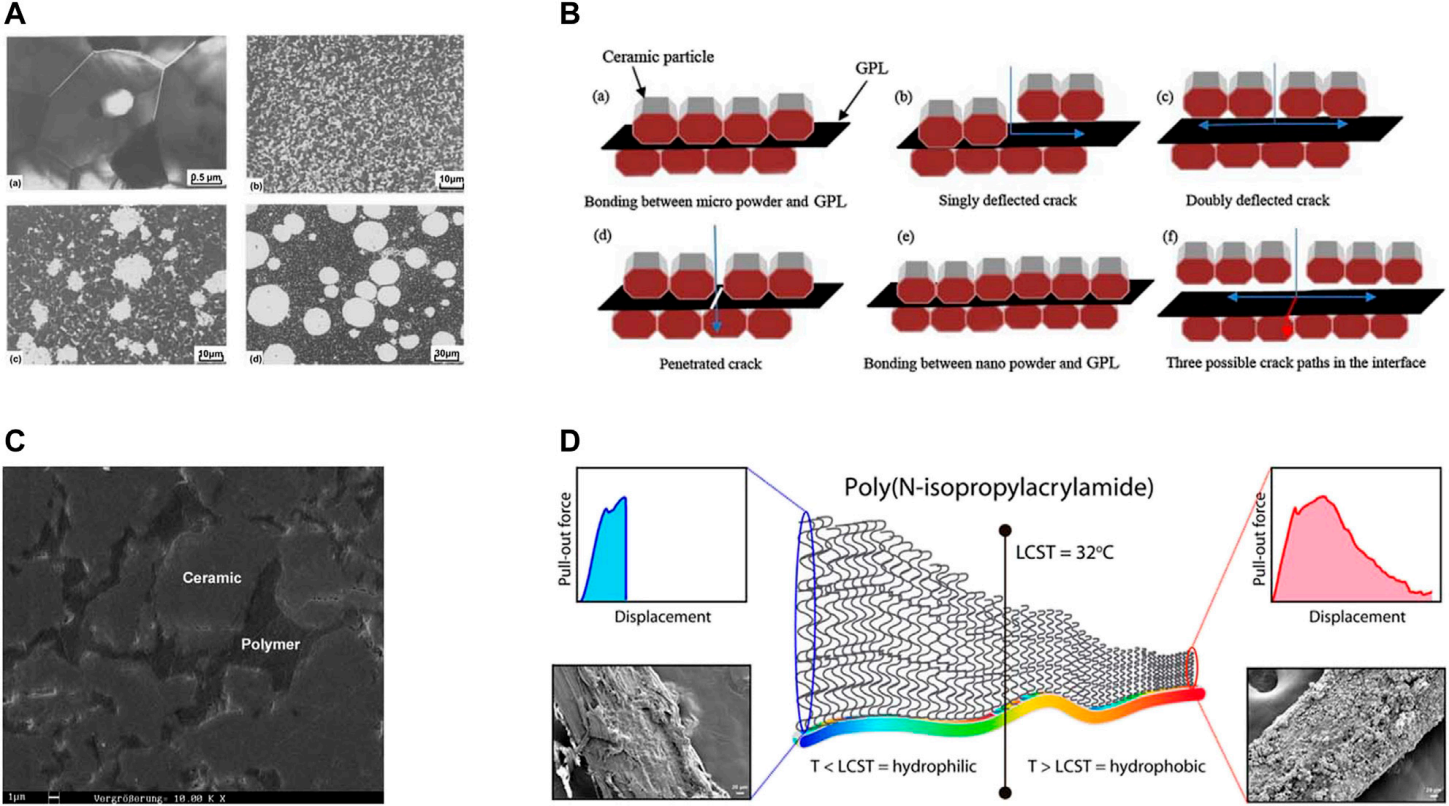
Adding reinforcing fillers as second phase to toughen ceramics. **(A)**, **(a)** transmission electron micrograph showing the microstructure for Al_2_O_3_ containing well-dispersed unstabilized ZrO_2_ particles; **(b–d)** scanning electron micrographs showing the microstructures of Al_2_O_3_ containing well-dispersed PSZ single crystals, TZP agglomerates and Al_2_O_3_-ZrO_2_ duplex structured composites, respectively (Wang and Stevens, 1989). **(B)** A schematic of the toughening mechanism in GPL toughened ceramic (Liu et al., 2012). **(C)** Polymer-infiltrated-ceramic-network structure (Coldea et al., 2013). **(D)** Thermoresponsive brushes facilitate effective reinforcement of calcium phosphate cements (Petre et al., 2019). PSZ, partially stabilized zirconia; TZP, tetragonal zirconia polycrastal; GPL, graphene platelet.

Concept of ductile-phase toughening is to introduce metal component, non-metal component or ductile polymers into a brittle matrix, like ceramic, to increase its toughness. The development of metal-toughened ceramic mainly associates with alumina (Al_2_O_3_) and zirconia (ZrO_2_), which could act as either matrix or reinforcement. And a large family of non-metal components used to toughen ceramic are nanocarbon materials, from one-dimensional to three-dimensional. Additionally, polymers infiltrated ceramics gain lots of attention recently. Apart from adding every type of fillers alone, combined materials of several types could also achieve the toughening purpose (Figure 2).

### 2.1 Metal Oxide and Metal Reinforcements

In 1976, ZrO_2_ was revealed to display high toughness (R. C. Garvie and Hannink, 1975), which ushered in a decade of exceptional ceramic toughening development, culminating in materials having toughness on the order of 20 MPa m^1/2^ (Evans, 1990). Undoped ZrO_2_ displays three phases at different temperature: the monoclinic (m) phase of undoped ZrO_2_ is thermodynamically stable at temperatures below 1,170°C; it is a tetragonal (t) phase from 1,170 to 2,370°C, and a cubic (c) phase above 2,370°C until the melt occurs at 2,706°C (French et al., 1994). Because the t-to-m phase transformation in zirconia may be used to toughen the material and enhance its mechanical properties, it has sparked a lot of technological interest in zirconia and composites incorporating zirconia (Heuer, 1987).

Using ZrO_2_ alone have the shortage of low-temperature degradation (LTD) (Kohorst et al., 2012), so Al_2_O_3_ are doped to exert synergistic effect. Two composite materials can be made in the ZrO_2_-Al_2_O_3_ system as follows: Al_2_O_3_ reinforced with ZrO_2_ particles and denominated as zirconia toughened alumina (ZTA), or ZrO_2_ reinforced with Al_2_O_3_ particles, named alumina toughened zirconia (ATZ) (Nevarez-Rascon et al., 2009). According to the findings of De Aza et al. (Ritchie, 1988; De Aza et al., 2002) and Gregori et al. (Gregori et al., 1999), the fracture toughness of the ceramic matrix material rose in both cases. ZTA can be considered a new generation of toughened ceramic, with toughness exceeding 12 MPa m^1/2^, compared to 3 MPa m^1/2^ for commercial alumina ceramic. The inclusion of discrete zirconia grains in the alumina matrix as a second phase allows the former to behave intrinsically, that is, to undergo the t-to-m transformation or to remain in the metastable tetragonal state after cooling of the composites from fabrication temperatures (Wang and Stevens, 1989). ZTA is microstructurally separated into four classes by Wang et al. (Figure 2A) (Wang and Stevens, 1989): (I) alumina with dispersed unstabilized zirconia (Figures 2A,a); (II) alumina with dispersed partially stabilized zirconia (PSZ) (Figures 2A,b); (III) alumina with PSZ agglomerates (Figures 2A,c); (IV) alumina-zirconia duplex structures (Figures 2A,d).

Except for these basic forms of ZrO_2_-Al_2_O_3_ materials, the shapes of both components could be altered, or other metal compounds could be added, to achieve better toughening effect. Aguilar-Elguézabal and Bocanegra-Bernal (2013) investigated the fracture toughness of an Al_2_O_3_(n)-70 wt% ZrO_2_ (ZrO_2_ with 3 mol% yttria, TZ-3Y)n nanocomposite with addition of 2.5% Al_2_O_3_ whiskers. The fracture toughness was enhanced 62, 28, and 7% over pure Al_2_O_3_, the composite without additions of whiskers, and pure TZ-3Y for medical applications, respectively. They achieved a maximum fracture toughness of 6.9 MPa m^1/2^ with an average grain size of 0.4 ± 0.17 μm, and found microcracking, crack deflection and phase transformation toughening mechanisms could contribute to the improvement of fracture toughness. Additionally, the Al_2_O_3_ whiskers did not affect other intrinsic properties during the toughening process. Santos et al. (2021) not only added Al_2_O_3_ in ZrO_2_, but also replaced the conventional tetragonal zirconia stabilized with 3 mol% yttria (3Y-TZP) with ceria and yttria stabilized zirconia, namely (Ce, Y)-TZP. And this new composite received higher fracture toughness, elastic modulus and hardness than those obtained for 3Y-TZP. In the meantime, its values of Weibull modulus (m > 10) and flexural strength (> 950 MPa) were similar to the 3Y-TZP ceramics, with high resistance to degradation in saliva, indicating adequate properties for dental application. They thought several different toughening mechanisms acting simultaneously in the material. Apart from the above ingredients of Santos et al.’s study, Gommeringer et al. (2019) reinforced 1Y6Ce-TZP materials with different amounts of alumina and/or strontium hexaalumina by slip casting and pressureless sintering at different temperatures. Then these materials underwent accession of their mechanical properties, microstructure, phase composition, and low-temperature degradation stability, which showed that they exhibited a high fracture resistance of 10–12 MPa m^1/2^. Partial substitution of the alumina dispersion by strontium hexaaluminate can improve the strength, while pres.erving toughness, hardness and low-temperature degradation resistance. The residual alumina provides grain growth inhibition. In summary, the improvement of structure or/and composition of the second phase is helpful to the toughening of ZrO_2_-Al_2_O_3_ ceramic.

ZrO_2_ and Al_2_O_3_ could also be added to toughen ceramics except for ZTA or ATZ. Gali et al. (2018) investigated ZrO_2_ toughened mica glass ceramics for dental restorations. It was recorded that mica glass ceramics with 20 wt% yttria stabilized zirconia (YSZ) reached Vickers hardness of 9.2 GPa, elastic modulus of 125 GPa, indentation toughness of 3.6 MPa m^1/2^, and chemical solubility of 30 g/cm^2^ (well below the permissible limit), respectively. The phenomenon of transformation toughening of YSZ was observed in enhancing the toughness properties of ceramic. Because of its osseointegration property, Calcium silicate (CaSiO_3_, CS), has been investigated as a bioactive biomaterial for bone tissue repair and replacement (Mehrali et al., 2014). Long et al. (2008) successfully fabricated β-CaSiO_3_/ZrO_2_ (3Y) nanocomposites via spark plasma sintering. Adding ZrO_2_ could enhance the phase transitional temperature of CaSiO_3_ and inhibit its phase transition. When the β- CaSiO_3_/ZrO_2_ mole ratio was 60/40, the fracture toughness and strength of the nanocomposites were as high as 4.08 MPa m^1/2^ and 395 MPa, respectively. Shuai et al. (2016) used zinc oxide whisker (ZnOw) to improve CaSO_4_/bioglass scaffolds which were prepared via selective laser sintering (SLS). And the ZnOw improved fracture toughness and compression strength significantly. The combination of several toughening mechanisms, including whiskers pull-out, crack bridging, crack deflection, and crack branching, might be attributed to the increase of mechanical characteristics. Shirazi et al., (2014) investigated the effect of Al_2_O_3_ on α-CaSiO_3_ ceramic and found that 15 wt% of Al_2_O_3_ addition at 1,250°C enhanced fracture toughness as well as hardness of CaSiO_3_.

Apart from ZrO_2_-Al_2_O_3_ system, other reinforcement-ceramic matrix systems have also been studied. Rasmussen et al. (2004) mixed core particles of cesium (Cs_2_O) -containing synthetic leucite with two cesium-free matrix porcelains, a commercial porcelain (VP) and a synthesized leucite-based porcelain (NP). The toughness of both types of composite materials was dependent on Cs_2_O content of the added core particles, and the ceramic containing 0.75 mol% Cs_2_O reached a maximum toughness. The possible toughening mechanism was transformation toughening. Luo et al. (2002) modified glass-infiltrated Al_2_O_3_ with 0.5 wt% MgO. And the fracture toughness of this composite (5.12 MPa m^1/2^) is obviously higher than the partially sintered Al_2_O_3_ (0.58 MPa m^1/2^), MgO-modified partially sintered Al_2_O_3_ (1.86 MPa m^1/2^), or glass-infiltrated Al_2_O_3_ (3.91 MPa m^1/2^).

Most above mentioned second phases for toughening are metal compounds rather than metals alone. Besides, the research of Fujieda et al. (2012) and Uno et al. (2013) showed that metal nanocomposites (NPs) could also be used to increase the fracture toughness of ceramic. Fujieda et al. (2012) found that the addition of silver (Ag) and platinum (Pt) NPs improved the mechanical properties, including the fracture toughness and the Young’s modulus, of commercial ceramic. The fracture toughness of AgNPs was higher than that of PtNPs. This could be due to the difference in positive compressive stress generation, which is projected to be greater for silver due to its higher coefficient of thermal expansion. Uno et al. (2013) researched the effects of adding AgNPs on the toughening of dental porcelain, Noritake Super (NS) Porcelain AAA. When the concentration of Ag in the solution was 500 and 1,000 ppm (Ag500 and Ag1000), toughness values of 1.54 MPa m^1/2^ and 1.51 MPa m^1/2^ were observed, respectively, which were higher than that of the control (1.36 MPa m^1/2^). The inclusion of Ag500 and Ag1000 NPs, on the other hand, resulted in a color shift. Some AgNPs remained nanoparticles, whereas others interacted with matrix elements and turned into silver ions. As a result of the ion exchange reaction and differential thermal expansion of the silver metal nanoparticles, a residual compressive stress was created. Swain S.K et al. (2016) developed a novel biocompatible β-tricalcium phosphate (β-TCP)-based composited by reinforcing ceramic matrix with 30 vol% of a biodegradable iron-magnesium (Fe-Mg) metallic phase, scilicet β-TCP-15Fe15 Mg and β-TCP-24Fe6 Mg (vol%) composites. Both these composites had increasing mechanical properties.

### 2.2 Non-Metal Reinforcements

#### 2.2.1 Nanacarbon

Nanocarbon materials have ever-increasing applications in ceramic toughening (Siddiqui et al., 2018), for displaying a special set of characteristics, namely high elasticity modulus, specific thermophysical, electrophysical and sorption properties (Falcao and Wudl, 2007). According to their spatial structures, nanocarbon materials can be classified as one-dimensional, two-dimensional and three-dimensional materials (Hirsch, 2010).

One of the representatives of one-dimensional carbon structures is carbon nanotubes (CNTs), possessing a tubular structure made by rolling single/multi-layer graphite sheets. Large specific surface areas (50–1,315 m^2^ g^−1^), a high aspect ratio (as they have their diameters at the nano-scale but their lengths, are, by contrast, at the micron-scale), high tensile strength, high resilience, and flexibility are its typical properties (Dong et al., 2012). On the basis of this physical structure, carbon nanotubes can be single-walled carbon nanotubes (SWNTs) (single tube)—or multi-walled carbon nanotubes (MWNTs) (concentric cylinders of carbon) (T. F. Kuo et al., 2001; Akasaka et al., 2006). Lahiri et al. (2010) synthesized MWNTs reinforced hydroxyapatite (HA) composite *via* spark plasma sintering. The fracture toughness and elastic modulus increased by 92 and 25%, respectively, compared to the HA matrix without CNT, which is due to the uniform distribution of 4 wt% CNTs in the HA matrix, good interfacial bonding and fine HA grain size. It was explained that interfacial shear strength and pull-out energy of MWNTs from HA contributed to toughening.

Graphene is a leading nanomaterial of two-dimensional carbonaceous materials (Allen et al., 2010). Compared to monolayer graphene, graphene platelets (GPLs), also referred as graphene nanoplatelets (GNPs), graphene nanosheets (GNS), or multilayer graphene nanosheets (MGN), are formed by several layers of graphene with thickness of up to100 nm (Siddiqui et al., 2018). GPLs have not only a large specific surface area, but also two dimensional high aspect ratio, as well as outstanding mechanical properties, which make them excellent potential nanofillers in composite materials (Liu Y et al., 2013). For dental ceramics, Liu et al. (2012) carried out a study on graphene platelet/zirconia-toughened alumina (GPL/ZTA) composites, which were sintered at different temperatures *via* spark plasma sintering. Measured by the single-edge notched beam method, it is found that at 1,550°C, GPL/ZTA composites reached almost full density, maximum fracture toughness (with 40% improvement) and hardness. Multi-toughening mechanisms, such as pull out, bridging and crack deflection, were observed. In the bone repair area, Mehrali et al. (2014) reinforced CaSiO_3_ ceramic with graphene nanoplatelets (GNPs) using hot isostatic pressing (HIP) method at 1,150°C. The homogeneous distribution of 1 wt% GNP in the CaSiO_3_ matrix, fine CaSiO_3_ grain size and high densification helped to increase the fracture toughness, brittleness index and hardness by 130, 40 and 30%, respectively, in contrast to the CaSiO_3_ matrix without GNPs. Similar to Liu et al. (2012), the toughening mechanisms also included crack bridging, pull-out, branching and deflection.

An important derivative of graphene is graphene oxide (GO), consisting of graphene sheets covered with oxygen-based functional groups (Raslan et al., 2020). Utilized in dental ceramic toughening, Zhang et al. (2020) distributed GO uniformly in 3Y-ZrO_2_ powders, forming the C-O-Zr bond during the sintering process. As a result, the fracture toughness increased 40.9% (8.95 ± 0.59 MPa m^1/2^) when adding 0.1 wt% GO, and the flexural strength improved up to 200% (1,489.96 ± 35.71 MPa) when adding 0.15 wt% GO, in comparison to raw 3Y-ZrO_2_ ceramics. The toughening mechanisms, namely crack deflection, crack bridging, and GO put-out are found.

#### 2.2.2 Polymer

Calcium phosphate cements (CPCs) are a class of injectable bone substitute bioceramics (Kucko et al., 2019). To toughen this kind of bioceramic, Kucko et al. (2019, 2020) added surface-modified poly (vinyl alcohol) (PVA) fibers to CPCs and improved toughness and flexural strength of CPCs by more than 435-fold and 3-fold, respectively. The fiber-matrix affinity is thought to be paramount in designing highly toughened CPCs. So, the surface of PVA fibers was modified to improve their hydrophilicity and affinity to the CPC matrix, which facilitated energy dissipation during fracture of CPCs. Furthermore, other crack-arresting mechanisms may also play a significant role in mechanically reinforcing CPCs, because the fracture toughness improved significantly even for CPCs reinforced with fibers of lengths greater than their critical fiber embedment length. The surface of PVA fibers could be further modified with environmentally responsive materials to improve their ceramic toughening effect. Petre et al. (2019) functionalized the surface of PVA fibers with thermoresponsive poly (N-isopropylacrylamide) brushes of tunable thickness to enhance simultaneously fiber dispersion and fiber-matrix affinity. At temperatures above their lower critical solution temperature of 32°C, these brushes shifted from hydrophilic to hydrophobic. This dual thermoresponsive shift favored fiber dispersion throughout the hydrophilic CPCs (at 21°C) and toughened these cements when reaching their hydrophobic state (at 37°C). And fibers at 37°C showed nearly double reinforcement efficacy than that of 21°C.

**FIGURE 3:**
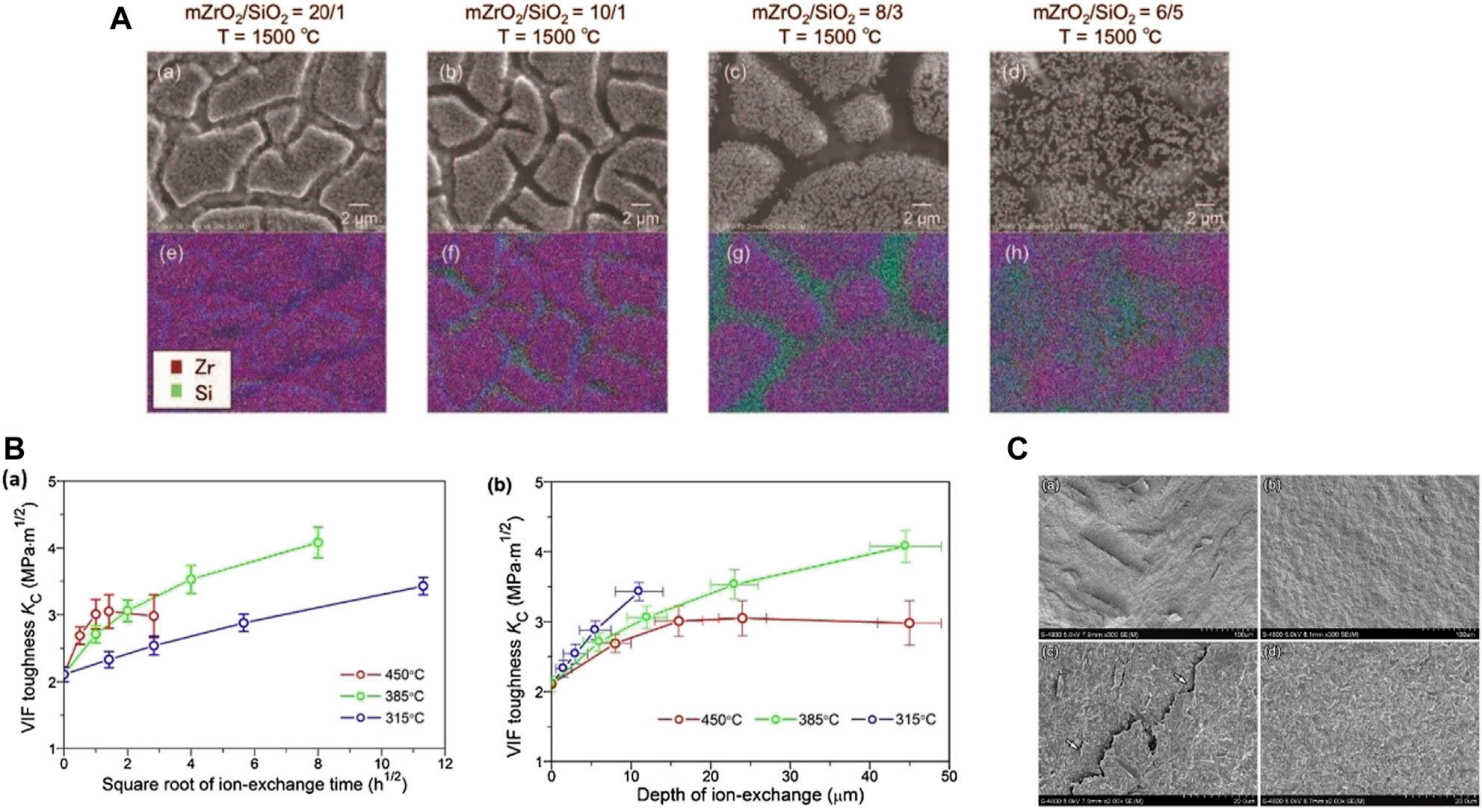
Toughening ceramics by coating, chemical and thermal surface treatments. **(A)** Monoclinic ZrO_2_ and SiO_2_ particle coating, toughening highly translucent ZrO_2_. **(a–d)** scanning electron micrographs, **(e–h)** Zr and Si elemental mapping of samples coated with mZrO_2_/SiO_2_ and heat treated at 1,500°C (Uno et al., 2020). **(B)** Ion-exchange toughened lithium disilicate glass-ceramic. Changing tendencies of VIF toughness of the glass-ceramic with increasing **(a)** the ion-exchange time and **(b)** the depth of Li^+^/Na^+^ exchange obtained, at 450, 385 and 315°C, respectively, in a pure NaNO_3_ bath (Li et al., 2020). **(C)** Scanning electron microscope fractographs of the lithium disilicate glass-ceramic: **(a,c)** in annealing state; **(b,d)** tempered in 250°C silicone oil. Reproduced with permission from Li et al. (2021). VIF, Vickers indentation fracture.

Different from conventional dispersed-filler toughened ceramics, a kind of dual-phase material consists of two continuous interpenetrating networks, namely polymer infiltrated ceramic network (PICN) material, has been developed to emulate the properties and physical properties of natural teeth (Mainjot et al., 2016). PINCs can be sorted as interpenetrating phase composites, which possess a three-dimensional interconnected geometry. The two continuous networks in PICNs are porous pre-sintered ceramic networks and infiltrated polymers (commonly resin for dental applications), which are shown in Figure 1 (Coldea et al., 2013). Crack propagation is often limited in PICNs due to interfacial crack deflection, which is caused by the existence of two linked phases. By bridging the cracks introduced to the other phase, the phase with the higher strain to failure improves fracture resistance (Horvitz et al., 2002; Nalla et al., 2003). Furthermore, Feng et al. (2003) found that the reinforcing phase could distribute stresses more efficiently in all directions in interpenetrating networks. Released in 2012, Vita Enamic (Vita Zahnfabrik, Bad Säckingen, Germany) is the successful representative of commercial PICN, consisting of 86 wt% or 75 vol% inorganic phase and 14 wt% or 25 vol% organic phase (Swain M. V. et al., 2016). Della Bona et al. (2014) examined the microstructure and fracture toughness (K_Ic_) of Vita Enamic, *via* scanning electron microscopy (SEM) and V-notched-beam test, respectively. The microstructure was shown in Figure 3 and the K_Ic_ was 1.09 ± 0.05 MPa m^1/2^. El Zhawi et al. (2016) tested the resistance to fatigue fracture and wear of Vita Enamic and used step-stress accelerated life testing to show that failure could only be found under very high loads (>1000 N). The result inferred that it was unlike for the Enamic posterior crowns to experience early clinical abrupt fractures, which may only happen under extremely high traumatic loads when the radius of the antagonist cusp was relatively small. Swain M. V et al. (2016) investigated seven kinds of PICN materials, whose single edge V-notched bend (SEVNB) fracture toughness varied from 0.82 to 4.94 MPa m^1/2^. And they indicated that factors influencing the extent of the crack included the residual stress in the resins, the bonding quality with the ceramic matrixes, the extent of polymeric conversion of the monomer or oligomer, as well as the yield stress and strain to failure of the bridging polymer. Li et al. (2017) fabricated a polymer-infiltrated zirconia ceramic for dental crown restoration, with zirconia network porosity varying from 46.3 to 34.7% and the relevant polymer content ranging from 18.4 wt% to 12.3 wt%. At pre-sintered temperature was 1,150°C, the facture toughness was 3.69 ± 0.15 MPa m^1/2^, about twice or three times higher than those of the polymer-infiltrated feldspar ceramics.

#### 2.2.3 Others

On the periodic table of the elements, silicon and carbon are both on the fourth group, which indicates that silicon may have similar effect as carbon and could also be used in ceramic toughening. Silicon nitride (Si_3_N_4_) is a kind of ceramics with high performance characterized by fracture toughness, high wear resistance and low coefficient of friction (Pezzotti, 2019). Pan et al. (2015) doped different content of Si_3_N_4_ in β-CaSiO_3_ ceramics at the sintering temperature ranging from 1,000°C to 1,150°C. Through being oxidized to form SiO_2_, Si_3_N_4_ can be successfully used as sintering additive. And with 3 wt% Si_3_N_4_ addition, the β-CaSiO_3_ ceramics sintered at 1,100°C got fracture toughness of 2.3 MPa m^1/2^, flexural strength of 157.2 MPa and hardness of 4.4 GPa, which was much higher than that of pure β-CaSiO_3_ ceramics (1.1 MPa m^1/2^, 41.1 MPa and 1.0 GPa).

In organic compounds, chirality is a very important property, which seems to also play a role in ceramic toughening materials. Moussa et al. (2020) showed that the addition of homochiral L-(+)-tartaric acid (Tar) increased the mechanical properties of brushite bioceramics by decreasing their crystal size, following the classic Hall-Petch strengthening effect; however, D-(-)-tartaric acid displayed the opposite effect. In comparison with brushite bioceramics without additives, adding L-(+)-Tar increased the fracture toughness (0.3  MPa m^1/2^) and compressive strength (26 MPa) of this ceramic composite by 62 and 33%, respectively.

### 2.3 Multi-Component Reinforcements

Both metal and non-metal reinforcements have their advantages and disadvantages. Metal fillers like ZrO_2_ or Al_2_O_3_ have esthetic color but unexpected LTD or lower strength, while non-metal fillers like nanocarbon materials possess high elasticity modulus but susceptible to high sintering temperature. Proper combination of metal and non-metal components could be a promising approach to toughen bioceramics. Cesar et al. (2005) modified six kinds of dental porcelains with the addition of leucite. Leucite (K_2_O·Al_2_O_3_·4SiO_2_) is a potassium–aluminum–silicate phase that can be incorporated into the dental porcelain in two ways: the incongruent melting of potash feldspar (K_2_O·Al_2_O_3_·6SiO_2_) or as a synthetic powder. For the materials investigated, the higher the leucite content in the porcelain, the higher the fracture toughness, implying that they would have better clinical performance. Xiang et al. (2007) fabricated mica-based glass-ceramics containing needle-like fluorapatites. They found that the ceramics with higher fluorapatite content (mainly containing CaO and P_2_O_5_) presented higher fracture toughness and Vickers hardness, due to a large amount of needle-like fluorapatite crystals and the fine microstructure. Luo et al. (2002) fabricated MgO-modified glass infiltrated Al_2_O_3_ for CAD/CAM. There are two main functions of glass infiltration: the first is to eliminate almost all porosities, which are prone to crack initiation; secondly, the difference in the coefficients of thermal expansion between Al_2_O_3_ and glass produces compressive stresses, further enhancing strength at the Al_2_O_3_-glass interface. Furthermore, MgO improved grain size uniformity, controlled grain growth, and promoted homogeneous wetting of Al_2_O_3_ grains by the liquid through an alternation in interfacial energies, allowing the manufacture of high-density ceramics.

## 3 Surface Modification of Ceramic

Surface modification mainly includes coating, chemical and thermal surface treatments (Kelly et al., 1996) (Figure 3).

Coating is also an important and widely used method in surface modification. Based on the toughening mechanism of strengthened glass, where the outer surface is compressed to impede breakage because of the outside stress, Uno et al. (2020) used a dispersion containing mZrO_2_ and SiO_2_ as a surface coating agent for partially stabilized zirconia (PSZ). When the crystal phase changed from tetragonal to monoclinic, the mZrO_2_ underwent volume change to generate a compressive stress layer on the material’s surface. The SiO_2_ serves as a binder to improve wettability and to accelerate the sintering of mZrO_2_.

Because many (but not all) ceramic and glass structures collapse due to surface imperfections, and surface compressive stresses must be exceeded before cracks can propagate, this approach permits treated structures to withstand higher loads before failing (Kelly et al., 1996). Ion-exchange has become a major method for chemical toughening, the mechanism of which could be attributed to “stuffing effect”——exchange of smaller alkaline metal ions in glasses with larger ones from molten salt baths can cause residual compressive stresses on the surfaces at temperatures below the glass transition temperatures (Tg), resulting in significant toughening of the glasses under the condition without sacrificing their light transmittance (translucency) (Li et al., 2020). This mechanism indicates that ion-exchange is suitable for ceramics containing glass (glass-ceramics), dual-phase materials consisting of glassy matrix and embedded crystalline phases (Deubener et al., 2018). They could combine the attractive properties of crystalline ceramics with those of glasses. Ion-exchange has been a popular way to strengthen monolithic silicate glasses containing alkaline oxides (Fillery and Lange, 2007; Jiang et al., 2017). In Li et al.’s study (Li et al., 2020), a multi-component lithium disilicate (LD) glass-ceramic with interlocking microstructure consisting of rod-like LD crystals and glassy matrix was ion-exchanged over wide temperature and time range in pure NaNO_3_ or mixed NaNO_3_ and KNO_3_ baths below the glass transition temperature. Moreover, an experimental dual ion exchange toughening treatment was developed for feldspathic porcelains that surpassed the toughening of a single ion treatment and survived air abrasion (Kelly et al., 1996).

Thermal treatment could also effectively toughen glass ceramics. Li et al. (2021) toughened lithium disilicate (LD) glass-ceramic by tempering processes, which were conducted by heating the bar-like and disc-like specimens to a temperature below the dynamic softening point, and then rapid cooling in silicone oil with different temperatures ranging from room-temperature to 300°C to regulate the cooling rate. The specimens were effectively toughened, but the bar-like specimen displayed obvious anisotropy in fracture toughness, attributed to the “edge cooling effect” of the specimens with uneven geometry.

Comparing the chemical and thermal toughening methods, the former has the particular benefit of efficiently toughening glass-ceramic products of nearly any geometry and thickness (Donald, 1989), which is critical for many types of medical devices with customized geometries and thicknesses. However, this toughening effect could be susceptible to contact damage during service (Gy, 2008), or it will be completely lost in clinical practice by surface grinding during the manufacture of devices, like dental restorations, owing to the small micron level case depth of ion-exchange (Fischer et al., 2008). On the contrary, the thermal tempering could create significantly deeper surface layers with residual compressive stresses (millimeter level), making the toughening effect less susceptible to contact damage (Fan et al., 2016). Unfortunately, it was observed that the residual stresses created by fast cooling were geometry-dependent, and that they might vary in different directions as a result of differences in heat exchange conditions caused by different geometries (Al-Amleh et al., 2014). Therefore, mechanical anisotropy of the tempered glass-ceramics would emerge from these geometry-dependent residual stresses, lowering their mechanical reliability.

## 4 Improving Manufacturing Processes

The fabrication of ceramics is concerned with powder preparation, green forming and the densification processes (Wang and Stevens, 1989): (I) powder preparation processes include mechanical mixing, sol-gel processes, partial chemical methods, chemical vapor deposition (CVD) process, rapid solidification, hydrothermal oxidation, wear of ZrO_2_ milling media, etc. (II) forming methods mainly involve regular sintering, spark plasma sintering (SPS) (Han et al., 2017), hot pressing, hot isostatic pressing and so on.

Considering optimization of powder preparation processes, grain sizes could affect mechanical properties of ceramics. Larger particles undergo the martensitic t-to-m transformation more quickly than smaller particles in partially stabilized ZrO_2_ and Al_2_O_3_-ZrO_2_ composites (Heuer et al., 1982). By decreasing the corresponding grain sizes and enhancing the homogeneity of the phase dispersion, the mechanical characteristics of ZrO_2_/Al_2_O_3_ dispersion ceramics may be significantly improved. Bartolomé et al. (2016) prepared nanoparticles with an intraparticular phase distribution of Zr_(1-x)_Al_x_O_(2-x/2)_ and (γ-,δ-)Al_2_O_3_ by the simultaneous gas phase condensation of CO_2_ laser co-vaporized (CoLAVA) zirconia and alumina raw powders. In general, the CoLAVA nanoparticles are spherically shaped, narrowly size-distributed, crystalline, and merely softly agglomerated by weak van der Waals forces. And fracture toughness of the CoLAVA composite significantly exceeds the ones of the wet mechanically mixed Al_2_O_3_/ZrO_2_ reference powder (WM) by 45%, and reaches levels of 6.8 MPa m^1/2^.

In addition to particle sizes, degree of crystallization also affects ceramic toughness, which could be referred to as crystallization toughening. Serbena et al. (2015) employed stoichiometric lithium disilicate glasses as a model system, which were crystallized using two-stage heat treatments that were carefully developed and controlled to yield varied crystallized volume fractions while maintaining a constant grain size of around 12 μm. The fracture toughness of a completely crystalline sample improves about fivefold, from 0.75 to 3.5 MPa m^1/2^. Crack deflection, crack bowing and trapping, and crack bridging are three methods that contribute to toughening.

Regarding the densification of materials, sintering methods influence the final properties and microstructure of the obtained material (Chaim, 2008; Nevarez-Rascon et al., 2009; Faga et al., 2012). Magnani and Brillante (2005) found that post-hot isostatic pressing treatment resulted in the formation of a small amount of monoclinic phase in Al_2_O_3_-ZrO_2_ composites that decreased fracture toughness. Gil-Flores et al. (2020) densified ATZ nanocomposites via nonconventional microwave sintering technology at relatively low temperatures (1,200 and 1,300°C). The results indicated that the density increased as the sintering temperature was higher, which lead to improved mechanical properties, reaching a maximum fracture toughness (5.7 ± 0.3 MPa m^1/2^) and hardness (18.4 ± 0.4 GPa).

## 5 Toughening Mechanisms

**FIGURE 4:**
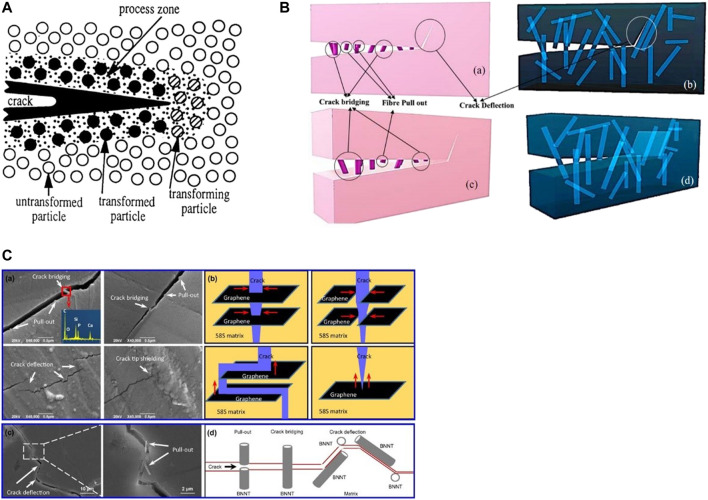
Toughening mechanisms in ceramic composites. **(A)** Representation of stress-induced phase transformation toughening process (Piconi and Maccauro, 1999). **(B)** A typical representation of strengthening and toughening by fibers. **(a,c)** represent two views of crack in a body being perturbed by fibers, **(b,d)** represents the see-through image of the body presented in **(a,c)** (Siddiqui et al., 2018). **(C)** Reinforcing mechanisms of low-dimensional nanomaterials (LDNs) in bioactive ceramics. **(a,c)** scanning electron micrographs and **(b,d)** schematic diagram showing the pull-out, crack bridging, crack deflection and crack tip shielding mechanisms (Gao et al., 2017).

In short, the major toughening mechanism of ceramics is to absorb energy of crack initiation or propagation to impede ceramic fracture. Different toughening methods have their specific mechanisms, but may also share similar mechanisms, vice versa, one toughening method typically contains several mechanisms at the same time (Figure 4).

### 5.1 Fine-Grained Toughening

The underlying toughening mechanism of controlling grain size is mainly fine-grained toughening. The reinforcement fillers located at the grain boundary provide a pinning effect, which can not only inhibit grain growth but also promote filler dispersion (Karthiselva et al., 2018). The toughening effect of fine-grain reinforcement is quite obvious according to the Hall-Petch formula. And the vital factors influencing the efficiency of fine-grained toughening include the content of second phase, the geometric shape, as well as the temperature and impurities (Liu et al., 2020).

### 5.2 Stress-Induced Phase Transformation Toughening

Transformation toughening mainly occurs in ceramics consisting of ZrO_2_, and could sometimes happen for other components as well. When an external tensile stress is applied to a crack tip, metastable tetragonal ZrO_2_ inclusions scattered in a ceramic matrix are known to convert to their thermodynamically stable monoclinic form (R. C. Garvie and Hannink, 1975; Lange, 1982). The stress to initiate transformation is lower than the fracture stress, and transformation happens preferentially and proceeds until the transformable t phase is exhausted (Hannink et al., 2000). Therefore, the phase transformation, which is accompanied by a volume expansion of 4% and shear strain of 6%, creates a compressive stress that slows and eventually stops the crack propagation, while the strain energy associated with any net shear component of the transformation strain in the transformation zone contributes an effective increase in the energy of fracture (Wang and Stevens, 1989). The contribution of stress-activated transformation, ∆K_cT_, to the toughness is usually expressed in the form

$$\Delta K_{cT}=\frac{\eta E^{*}e_{T}V_{f}h^{1/2}}{1-\nu }$$ (2)

Where η is a factor depending on the zone shape at the crack tip and the nature of the stress field in that zone, E^∗^ the effective modulus of the material, e_T_ the dilatational strain, V_f_ the transformed volume fraction of the particles, h the width of the transformation zone from the crack surface, and νthe Poisson ratio (Hannink et al., 2000).

### 5.3 Microcrack Toughening

Microcracks in ceramic materials can be subdivided as residual microcracks and stress-induced microcracks. Take the ZrO_2_ ceramic as an example, the residual microcracks are those caused by the volume expansion and shear strain associated with t-to-m phase transformation mentioned in section above, while the stress-induced microcracks are from the volume expansion and shear strain associated with the subsequent stress-induced transformation during fracture process. During phase transformation, tangential stresses are generated around the transformed m-ZrO_2_ particles. Microcrack toughening mainly relies on two shielding sources. One is the main-crack and microcrack interaction which causes stress redistribution ahead of the main-crack tip and thus lowers the continuum stiffness of the microcracked material. The other is the stress redistribution at the main-crack tip due to the release of residual stress when a microcrack is nucleated (Hutchinson, 1987). Microcracks form at the intersections of the fillers and the matrix as a result of this, which can absorb fracture energy by extending in the stress field of a propagating crack or deflecting the propagating crack (Wang and Stevens, 1989). Microcracks behind the main-crack tip provide the most shielding, while microcracks ahead of the main-crack tip contribute no shielding effect. What’s more, compressive normal residual stress may attribute to an anti-shielding effect at the main-crack tip, which should be paid attention for the design of ceramic materials (Chen, 2005).

### 5.4 Crack Deflection and Crack Bifurcation

Crack deflection refers to the condition that the reinforcement fillers will generally deflect the crack at a certain angle when the crack propagates in the ceramic matrix and meets fillers (Zou et al., 2018). So the fracture energy of crack propagating is consumed and the rate of crack propagating is reduced, as a result, the fracture toughness is increased (Zou et al., 2018). Specifically, deflection toughening occurs whenever interaction between the crack front and the minor phase produces a non-planar crack, subject to a stress intensity lower than that experienced by the corresponding planar crack. And the non-planar crack arises either from the existence of weakened interfaces or from residual strains present in the material. Considering shape, the rod of high aspect ration is the most effective morphology to deflect propagating cracks, which could account for four-fold increases in fracture toughness (Faber and Evans, 1983).

Crack bifurcation occurs at the matrix-filler interface. The primary crack runs vertically, whereas the bifurcation crack runs parallel to the matrix-filler interface. When a micro-crack forms at the end of the interface and can be deflected at 90° to extend to the ceramic matrix, the bifurcation crack is ended, which helps stress propagation at the corner. The new fracture then spreads parallel to the primary crack (Cheng et al., 2018).

### 5.5 Crack Bridging and Pull-Out

Take advantage of crack bridging, several types of discontinuous reinforcing fillers have been successfully applied to form toughened ceramics including second-phase whiskers (Becher, 1990), platelets (Becher, 1991), as well as elongated, plate-like, and large matrix grains (Himsolt et al., 1979; Mussler et al., 1982). Crack bridging happens during ceramic cracking. The two ends of the reinforcement filler link the crack surface and begin to distort as the distance between the two crack walls grows, which imposes a closure force on the crack. The two segments of fillers are tightly linked to the matrix throughout the process and consume the crack propagation energy.

Before reaching the reinforcement’s deformation limit, a pull-out may occur, consuming the fracture energy, or filler-matrix debonding with crack bridging may occur. Defects in this mechanism alter the mobility of fillers, which in turn affects energy dissipation (Liu et al., 2020). The extent of pullout of discontinuous reinforcement is commonly quite limited, which is due both to the short length of such phases and the fact that bonding and clamping stresses often discourage pull out. However, pullout cannot be ignored as even short pull-out lengths contribute to the toughness achieved (Li, 2012).

## 6 Current Challenges and Future Outlook

Although large amount of toughening strategies have been proposed for biomedical ceramic, there still exists challenges needed to be overcome, such as biodegradability and biocompatibility, as well as trade-off between toughness and strength. These challenges indicate that there is broad developing space of ceramic toughening for biomedical use, and future outlook is put forward to shed light on this attractive research field.

### 6.1 Influence of Fracture Toughness of Ceramic on Degradability, *In Vitro* and *In Vivo* Performance

Biomedical applications require ceramic materials with excellent biocompatibility to ensure device acceptance and patient safety, which is the prior recommend compared with toughness. First, no short- or long-term cytotoxicity is expected for biomaterials. Nieto et al. (2017) reviewed studies on graphene reinforced ceramic matrix composites (GNP-CMCs) and found all studies showed no cytotoxic effects induced by GNPs. After evaluating the long-term biocompatibility of a new ZTA, mainly in terms of DNA damage, mutagenicity and cancerogenetic potential in mammalian cells, Maccauro et al. (2009) found no long-term carcinogenic effect. Besides, toughening ceramic strategies should not affect the function of cells. In *in vitro* tests, the β-TCP-15Fe15Mg composite fabricated by Swain S. K et al. (2016) was shown to support the attachment and proliferation of osteoblast and endothelial cells and the cells exhibited characteristic markers for bone formation and angiogenesis, respectively. For the second phase addition strategy, proper concentration of reinforcement is a critical factor to be controlled. According to the evaluation of Zhang et al. (2019), hydroxyapatite ceramic with 3 wt% ZrO_2_ showed good cell viability and no cytotoxicity, and the mouse bone mesenchymal stem cells continued to proliferate on the ceramic surface observed for 5 days. Shuai et al. (2016) doped ZnO whisker (ZnOw) in the ceramic matrix, and cells attachment, extension and interconnection behavior of MG63 cells were enhanced with the ZnOw increasing from 1 wt% to 5 wt%. However, when ZnOw were further increased to 10 wt%, a sharp decrease of cell expansion degree was observed.

Biocompatibility of toughened ceramic has been studied mostly in terms of *in vitro* cytocompatibility, but fewer *in vivo* analysis has been conducted, which is a vital process before being considered for clinical applications. Petre et al. (2019) investigated the *in vivo* biocompatibility of both resorbable and nonresorbable PVA fiber-reinforced calcium phosphate cements. And these fibers were well dispered in the matrix without inducing inflammatory responses or other adverse reactions. Moreover, the inclusion of PVA fibers did not negatively affect the ingrowth of new bone into the PVA fiber-reinforced cements. The *in vivo* test of Kucko et al. (2019) also showed that PVA fibers do not compromise the excellent osteocompatibility of calcium phosphate cements. Nevertheless, further *in vivo* studies should be performed to investigate the biocompatibility of various types of toughened ceramics in more detail.

Degradability is significant for biomaterials like bone substitutes, which determines the final repair effect of bio-implants; while biomaterials for other applications, such as dental restorations or artificial joint, require to be stable rather than degradable. Therefore, most degradability tests of toughened ceramic are conducted in bone substitute ceramic composites. Significantly influenced by mechanical properties of biomaterials, degradability is mostly tested by soaking materials in the simulated body fluid (SBF) (Xiang et al., 2007). Toughening strategies, especially second phase addition may change the degradation rate and degradability. Li et al. (2015) found that the addition of ZnO or ZrO_2_ decreased the degradation rate, and improved the degradability of wollastonite. Similarly, Feng et al. (2014) observed the degradation rate of calcium silicate ceramic scaffolds decreased with the increase of hydroxyapatite whisker content. Moreover, toughened bone substitute ceramic possesses the apatite-forming ability in SBF, and osteoblast-like cells spread well on the scaffolds and proliferated with increasing culture time (Feng et al., 2014; Mehrali et al., 2014; Li et al., 2015). However, soaking in the SBF tests only the *in vitro* bio-degradability, and *in vivo* degradability should also be increasingly studied in the future.

### 6.2 Trade-Off Between Toughness and Strength, as Well as Esthetic and Function

Toughness and strength are typically exclusive properties, which means the improvement of toughness usually comes with a decrease in strength (Launey and Ritchie, 2009). If the decrease of strength is within proper range, the toughening methods are still acceptable, because of the following reasons. On the one hand, the strength of most ceramic is high enough to satisfy clinical requirements. On the other hand, the best strength is not the highest but the strength that matches those of the human tissues. Therefore, toughening is a more essential issue than strengthening for ceramics of biomedical applications and receives extensive attentions. Toughening strategies from second phase addition, surface modification to manufacturing process improvement have been studied to solve this problem. Considering different clinical application scenarios, ceramics for biomedical applications could be classified as esthetic-demanding and non-esthetic-demanding: dental restorations are the representatives of the former type of ceramic, in which esthetic sometimes is even paid more attention than toughness; while the latter, such as dental implants and artificial joints which could not be seen, mainly requires promising mechanical properties, especially toughness. What’s more, studies concerning esthetic, including color and translucency, are still limited. Adding reinforcing fillers as second phase to ceramic matrix is one of the most widely used strategies, and even commercial ceramic products have been fabricated based on this strategy; in contrast, surface modification strategies gain less studies and applications. Toughening based on ZrO_2_-Al_2_O_3_ system or mica glass ceramics (Gali and Kumar, 2019), and using ion-exchange (chemical methods) tends to preserve excellent esthetics, while adding colorful fillers like AgNPs (Fujieda et al., 2012) would affect ceramic color. For non-esthetic demanding applications, high toughness is the first consideration, which is fine achieved by fiber/whisker-shape (Aguilar-Elguézabal and Bocanegra-Bernal, 2013) or nanocarbon adding (Siddiqui et al., 2018).

### 6.3 Other Challenges

The ZrO_2_-Al_2_O_3_ system is well studied and used in clinic, such as dental restorations and implants, as well as femoral heads and hip joints. Although the addition of Al_2_O_3_ helps to improve the problem of low-temperature degradation (LTD) of ZrO_2_, it should still be paid enough attention during application, because most medical devices are applied in humid body environment which promotes degradation (Nguyen et al., 2009; Borchers et al., 2010). LTD refers to the t-to-m transformation of ZrO_2_ induced by hydrothermal aging in the humid environments, and with LTD, the energy barrier for t-to-m transformation is decreased due to incorporation of water constituents into zirconia lattice (Guo, 2004). Non-metal materials, especially nanocarbon materials, show their potential as reinforcement fillers due to excellent mechanical properties. Nevertheless, the application of nanocarbon materials still faces some challenges. Firstly, it’s difficult for nanocarbon to disperse in ceramic matrix due to its large specific surface area, surface energy, van der Waals forces caused by the intermolecular electrical dipole moment, and interactions between functional groups, as well as easy aggregation and entanglement properties (Liang et al., 2018). Secondly, the interface invasion between nanocarbon and ceramic is regarded unsuitable due to the differences in surface tension and density. Furthermore, it is difficult to determine the temperature during sintering, because nanocarbon can be destroyed at high temperatures when interface bonding is diminished. What’s more, measurement of ceramic matrix composites with nanocarbon is not homogeneous, for the composites are extremely anisotropic (Liu et al., 2020). Therefore, combination of different types of second phase materials may be a possible approach to exert synergistic effect and, in the meantime, diminish their disadvantages.

In addition to the type of second phase, the complex transition zone between ceramic matrix and reinforcement fillers, namely the interface, also play an important role on toughening, which is the basis of toughening mechanisms, such as pull-out and crack bridging. Proper interface strength is neither too weak nor too strong, so that the toughening mechanisms could dissipate energy during the loading process appropriately (Liu J et al., 2013; Ramirez et al., 2014; Ahmad et al., 2015).

De Aza et al. (2002) pointed out that the idea of a well-defined stress limit, toughness (K_IC_), must incorporate the concept of a threshold (K_I0_) under which crack propagation does not occur. The K_IC_ usually used only represents the resistance to fast fracture. However, ceramic materials are susceptible to slow crack propagation at K_I_ values under K_IC_, which is attributed to stress assisted corrosion at the crack tip, or any pre-existing defect in ceramic. The combined effect of high stresses at the crack tip and the presence of water or body fluid induce crack propagation in a subcritial manner (Chevalier et al., 1999). This slow crack propagation threshold may represent an intrinsic property of ceramic that gives information of its mechanical properties more realistic than the widely used toughenss, which represents only fast crack growth. The concept of slow crack propagation reminds us to pay attention to evaluations of mechanical properties, which may overestimate the behavior of ceramics.

### 6.4 Future Outlook

Microscopic building blocks, weak interfaces, and architecture are responsible for the extraordinary qualities of highly mineralized natural materials. Bio-inspired concepts could pave the way for new techniques to improve the toughness of brittle materials with enticing features but limited uses due to their brittleness (Wondraczek et al., 2011). These new materials show that bio-inspired techniques can be used to achieve both strength and toughness, which are normally mutually exclusive qualities (Ritchie, 2011). Nature transforms brittleness into toughness through three “overarching features”: (I) stiff and hard construction blocks are separated by (II) weaker interfaces, which are organized in (III) specialized architectures (Mirkhalaf et al., 2014). It may seem contradictory to make a material tougher by adding weak interfaces, but it appears to be a ubiquitous and powerful method in natural materials. Mirkhalaf et al. (2014) used three-dimensional laser engraving to create weak interfaces inside the bulk of glass and then infiltrating the interfaces with polyurethane. The crack is channeled towards toughening configurations by the weak interfaces, which obstructs its propagation. The technology produces a bio-inspired glass that is 200 times tougher than non-engraved, “intact” glass (namely bulk glass, which was not engraved, but that is not devoid of the surface defects typical to glass exposed to air). Glass and ceramic are both brittle materials, therefore, above mentioned bio-inspired method could also be attempted in ceramics. These bio-inspired concepts have opened new pathways to improving the toughness of ceramics, and in the recent years, increasing numbers of synthetic composites inspired from biological materials have emerged (Munch et al., 2008; Espinosa et al., 2011). This is one of the development directions of the future, with still limited researches, which calls for more investigating efforts.

## 7 Conclusion

Ceramic encounters limitations during biomedical applications due to its brittleness. Various extrinsic toughening strategies have been proposed to deal with this problem, including adding reinforcing fillers as second phase, surface modification and manufacture processes optimization. Proper concentration of metal or/and non-metal components, with different shapes, could both be added in ceramic matrix to enhance toughness, which is the mainstream of ceramic toughening, especially in the biomedical area. Apart from traditional dispersed-filler composites, dual phase interpenetrating networks and bio-inspired “block and mortar” architectures have also been developed to achieve better toughening effects. Surface coating, chemical and thermal toughening approaches could influence some depth of superficial layer to impede ceramic breakage, but each exhibit limitations. Furthermore, preparation of raw materials and forming methods of ceramic should be improved, as well. Underlying toughening mechanisms of these approaches include fine-grained toughening, stress-induced phase transformation toughening, microcrack toughening, crack deflection and bifurcation, and crack bridging and pull-out. In the future, effective combination of several toughening strategies and mechanisms should be the potential direction to extend the application scope of ceramic in biomedicine.

## Glossary

**Al**_**2**_ **O**_**3**_: alumina
AgNPs: silver nanoparticles
β-TCP: beta-tricalcium phosphate
BPO: benzoyl peroxide
Bis-GMA: Bisphenol A glycidyl dimethacrylate
**Cs**_**2**_ **O**: cesium oxide
(Ce,Y)-TZP: cerium oxide (CeO_2_) and yttrium oxide (Y_2_O_3_) stabilized zirconia (ZrO_2_)
**CaSiO**_**3**_: calcium silicate
**CaSO**_**4**_: calcium sulfate
CNT: carbon nanotube
CPCs: calcium phosphate cements
GNP: graphene nanoplatelets
GO: graphene oxide
GPL: graphene platelet
HA: hydroxyapatite
HIP: hot isostatic pressing
IF: indentation fracture method
**K**_**lc**_: fracture toughness
L-(+)-Tar: homochiral L-(+)-tartaric acid
NS: Noritake Super Porcelain AAA (Noritake Dental Supply Co., Nagoya, Japan)
PtNPs: platinum nanoparticles
PVA: poly (vinyl alcohol)
PBS: phosphate-buffered saline solution
PNIPAM: poly(N-isopropylacrylamide)
PICN: polymer infiltrated ceramic network
SPS: spark plasma sintering
SA6: strontium hexaaluminate
SLS: selective laser sintering
SENB: single-edge notched beam
**Si**_**3**_ **N**_**4**_: silicon nitride
SEVNB: single-edge V-notched beam
SEPB: single-edge precracked beam
MgO: magnesium oxide
TEGDMA: Tri(ethyleneglycol) dimethacrylate
WOF: work of fracture
3Y-TZP: 3 mol% yttria stabilized zirconia
1Y6Ce-TZP: 1 mol% Y_2_O_3_ + 6 mol% CeO_2_ stabilized ZrO_2_
ZnOw: zinc oxide whisker
ZTA: zirconia-toughened alumina
**ZrO**_**2**_: zirconia
